# Postoperative pain management satisfaction and its determinants among orthopedic patients in Northwest Ethiopia: a multicenter cross-sectional study

**DOI:** 10.1038/s41598-026-48420-7

**Published:** 2026-04-17

**Authors:** Temesgen Birlie Asmare, Amanuel Sisay Endeshaw, Negesse Zurbachew Gobezie, Getachew Mekete Deress, Kumlachew Geta Belete, Elias Alemayehu Worku, Biruk Demissie, Banchiayehu Alebachew Hailu, Kaletsidk Dessalegn Mossie, Yibeltal Yigzaw, Gezahagn Demsu Gedefaw, Denberu Eshetie Adane, Tewodros Wossen Gemechu, Habtie Bantider Wubet

**Affiliations:** 1 Department of Anesthesia, School of Medicine, College of Health Sciences, Debre Tabor University, Debre Tabor, Ethiopia; 2 Department of Anesthesia, College of Medicine and Health Science, Bahir Dar University, Bahir Dar, Ethiopia; 3 Department of Anesthesia, College of Medicine and Health Sciences, University of Gondar, Gondar, Ethiopia; 4 Department of Environmental Health, College of Health Science, Debre Tabor University, Debre Tabor, Ethiopia; 5 Department of Public Health, College of Health Science, Debre Tabor University, Debre Tabor, Ethiopia; 6 Department of Neonatal Health Nursing, School of Nursing, College of Medicine and Health Sciences, and Specialized Hospital, University of Gondar, Gondar, Ethiopia; 7 Department of Epidemiology, Debre Tabor Comprehensive Specialized Hospital, Debre Tabor, Ethiopia

**Keywords:** Postoperative pain, Patient’s satisfaction, Orthopedic surgery, Ethiopia, Diseases, Health care, Medical research, Risk factors

## Abstract

Postoperative pain is a common and distressing complication following surgery, particularly among orthopedic patients, due to extensive tissue injury and invasive procedures. Poorly managed POP can delay recovery, prolong hospital stays, increase complications, and reduce patient satisfaction. Despite the known burden, evidence on patient satisfaction with postoperative pain management among adult orthopedic patients in Ethiopia remains limited. This study aimed to assess patient satisfaction with postoperative pain management and its associated factors among adult orthopedic surgery patients at comprehensive specialized hospitals in the Amhara region from September 17 to December 13, 2025. A multicenter, institutional-based cross-sectional study was conducted among adult orthopedic surgical patients. A total of 423 participants were recruited using a consecutive sampling technique. Data were collected via semi-structured questionnaires adapted from validated tools and supplemented with locally relevant variables. Descriptive statistics were used to summarize participant characteristics, and bi-variable and multivariable logistic regression analyses were performed to identify factors associated with satisfaction. Adjusted odds ratios (AOR) with 95% confidence intervals (CI) were used to determine the strength of associations, with *p*-values < 0.05 considered statistically significant. Among the 423 participants, 79.4% (95% CI 75.30, 83.03) reported satisfaction with postoperative pain management. Factors significantly associated with higher satisfaction included undergoing elective surgery (AOR = 2.92, 95% CI 1.58–5.40), having no or low preoperative anxiety (AOR = 2.98, 95% CI 1.67–5.34), receiving preoperative analgesia (AOR = 2.39, 95% CI 1.29–4.40), shorter surgical duration (≤ 1 h) (AOR = 3.71, 95% CI 1.11–12.37), and use of peripheral nerve block-based postoperative analgesia (AOR = 2.98, 95% CI 1.27–7.02). Patient satisfaction with postoperative pain management was moderately high and significantly associated with several factors, including anxiety, preoperative analgesia, and perioperative practices such as elective surgery, shorter procedures, and the use of peripheral nerve block analgesia. To enhance postoperative pain control and patient satisfaction, healthcare facilities may consider prioritizing preoperative anxiety management, routine preoperative analgesia, and the consistent use of peripheral nerve block-based analgesia. In resource-limited settings, landmark-based techniques with appropriate provider training may support safe and effective implementation.

## Introduction

### Background

Postoperative pain (POP) is acute pain resulting from surgical trauma, accompanied by inflammatory responses and afferent neuronal stimulation that generate unpleasant sensory, emotional, and cognitive experiences^1^. Pain is among the most frequently reported postoperative complaints^2^.

Orthopedic surgical patients are particularly susceptible to severe postoperative pain due to extensive tissue injury, the invasive nature of procedures involving bones, joints, and muscles, inadequate pain assessment, and suboptimal use of analgesics^3^. However, postoperative pain is commonly under-assessed and inadequately treated, and understanding patients’ perspectives on pain management is essential for identifying gaps and improving the quality of surgical care^4^.

Globally, postoperative pain is one of the most common complications following surgery, affecting more than 47% of surgical patients^5^. In the United States, up to 80% of patients report dissatisfaction with pain management following orthopedic surgery^6^. The burden is even greater in Africa, where resource limitations and systemic challenges contribute to substantially higher rates of postoperative pain compared to developed countries^2^. Evidence indicates that up to 95.2% of African surgical patients experience postoperative pain, highlighting the persistent difficulty of achieving effective pain control in low-resource settings^7^. Inadequate management of POP remains a recurrent source of patient dissatisfaction and is associated with adverse clinical outcomes^8^.

Poorly controlled postoperative pain interferes with daily activities, sleep, and emotional well-being and is frequently accompanied by inadequate pain assessment and minimal patient involvement in pain management decisions^9^. In orthopedic surgery, postoperative pain is often more severe and prolonged than in other procedures, leading to delayed mobilization, prolonged hospital stays, and an increased risk of complications when pain is poorly managed^10^. Severe POP may delay recovery, cause physical and psychological harm, and progress to chronic pain syndromes, thereby increasing long-term morbidity^11^. Inadequate pain control is also associated with increased healthcare costs, higher readmission rates, prolonged hospitalization, and, in severe cases, increased mortality^12,13^.

The primary goal of postoperative pain management is to minimize the negative consequences of acute pain, promote early mobilization, and facilitate a smooth return to normal function^14^. Effective pain control improves patient comfort and satisfaction, reduces postoperative complications, shortens hospital stays, enhances recovery of physical activity, and improves overall surgical outcomes^15^. Although the introduction of acute pain teams has improved pain management in many acute care settings, pain control remains a significant and recurring source of patient dissatisfaction^2,9^.

Patient satisfaction is defined as the degree to which patients perceive that their expectations of healthcare services have been met^16^. Patient satisfaction with postoperative pain management is influenced by multiple factors, including age, gender, preoperative expectations, adequacy of preoperative information, American Societies of Anesthesiologist (ASA) status, use of preoperative analgesics, type of anesthesia, type and duration of surgery, healthcare provider communication, and perceived effectiveness of pain relief^6,17^.

Despite the substantial burden of postoperative pain, evidence regarding patient satisfaction with postoperative pain management—particularly among orthopedic surgical patients—remains limited in the Ethiopian context. Orthopedic procedures are frequently associated with higher postoperative pain intensity due to extensive musculoskeletal tissue trauma. However, most previous studies conducted in Ethiopia have either focused on mixed surgical populations or were limited to single an institution, which restricts their applicability to orthopedic-specific settings. Furthermore, important determinants of patient satisfaction, including preoperative anxiety, perioperative analgesic strategies, and the use of regional anesthesia techniques, have not been sufficiently explored within resource-limited healthcare environments. Addressing these gaps is important because patient satisfaction reflects both the effectiveness and quality of pain management services. Therefore, this multicenter study was designed to assess the magnitude of patient satisfaction with postoperative pain management and identify its associated factors among adult orthopedic surgical patients in comprehensive specialized hospitals in Northwest Ethiopia. By providing context-specific evidence, the findings may support clinicians, hospital administrators, and health authorities in identifying areas for improvement and strengthening perioperative pain management practices.

## Objective

To assess satisfaction with postoperative pain management and its associated factors in adult patients who underwent orthopedic surgery at the Comprehensive Specialized Hospital in the Amhara region of Northwest Ethiopia from September 17 to December 13, 2025.

## Method and material

### Study area

The study was conducted at three tertiary hospitals in Northwest Ethiopia. Debre Tabor Comprehensive Specialized Hospital (DTCSH), located in Debre Tabor city, South Gondar zone, is 103 km from Bahir Dar and 666 km from Addis Ababa. Established in 1953 by a Norwegian missionary, it was upgraded to a comprehensive specialized hospital in 2020. The orthopedic ward has 16 beds with an average monthly admission of 85 patients, and the hospital performs orthopedic surgeries for 79 patients per month across two operating rooms.

Tibebe Ghion Specialized Hospital (TGSH), in Bahir Dar, 565 km from Addis Ababa, was established in 2019. Its orthopedic ward has 54 beds with an average monthly admission of 180 patients, performing 164 orthopedic surgeries per month in two operating rooms.

University of Gondar Comprehensive Specialized Hospital (UoGCSH), in Gondar city, 663 km northwest of Addis Ababa, is the oldest hospital in the area. The orthopedic ward has 37 beds (plus 27 trauma beds), with an average monthly admission of 123 patients, and performs 112 orthopedic surgeries per month in three operating rooms, one of which is shared with general surgery.

### Study design and period

A multi-center, institutional-based cross-sectional study was conducted from September 17 to December 13, 2025.

### Eligibility criteria

Patients aged 18 years or older who underwent orthopedic surgery were included in the study. Exclusions were made for individuals who were unconscious or unable to communicate during the data collection period, as well as for patients who underwent surgery under local anesthesia without postoperative analgesic follow-up. Additionally, patients with hearing, speech, or language barriers that hindered effective communication and who did not have a translator were excluded to ensure accurate understanding of the interview questions and reliability of the responses. Patients with known psychiatric disorders were also excluded because these conditions may affect perception, interpretation, and reporting of satisfaction and pain experiences.

### Sample size determination

The sample size (*n*) was calculated using the single population proportion formula for cross-sectional surveys, applying a 95% confidence interval and a 5% margin of error^18^. Due to the lack of prior studies in our country, we assumed a 50% satisfaction rate with postoperative pain management among adult patients who underwent orthopedic surgery. The formula was applied as follows:

$${\mathrm{n}} = \frac{{\left( {{\mathrm{Z}}{ alpha }/2} \right)^{2} {\mathrm{P}}\left( {{1} - {\mathrm{P}}} \right)}}{{{\mathrm{d}}^{2} }}$$

where (n) is the estimated sample size, (P) represents the estimated satisfaction of POP management in adult patients who underwent orthopedic surgery, set at 50%. (Zα/2) is the critical value at the 95% level of significance, equal to 1.96. (d) is the margin of error, set at 5%

$${\mathrm{n}} = \frac{{(1.96)^{2} *0.5*(0.5)}}{{(0.05)^{2} }}$$

n = 384; after accounting for a 10% non-response rate, the final sample size was 423.

### Sampling techniques

A consecutive sampling technique was used to obtain the required sample size.

### Study variables

#### Dependent variables

Satisfaction with POP management (Satisfied/Not satisfied).

#### Independent variables

Socio demographic factors (age, sex, educational status, marital status, occupation, and residency). Clinical related factors (ASA status, preoperative anxiety, history of substance use, history of chronic pain, urgency of surgery, types of procedure, types of analgesia given, types of anesthesia and duration of surgery).

### Operational definition

*Patient satisfaction* Patient satisfaction with postoperative pain management was assessed using a structured questionnaire adapted from the Acute Pain Management Service (APMS) patient satisfaction tool, comprising 10 items: six rated on a five-point Likert scale and four with binary response options (Yes/No). Each item was scored, and a total satisfaction score was computed for each participant. The level of satisfaction was determined using the demarcation threshold formula: = ((total highest score − total lowest score)/2) + total lowest scores^19^. For this study, the minimum possible total score was 6, the maximum 34, and the calculated cutoff point was 20. Participants with a total score ≥ 20 were classified as satisfied, while those scoring < 20 were considered not satisfied.

*Pain* The severity of pain is classified based on Numerical rating scale as 0 = no pain, 1–3 = mild pain, 4–6 = moderate pain, 7–10 = severe pain^20^.

*Anxiety* Anxiety was assessed using the short version of the State Anxiety Inventory, which includes six components. To calculate the score, sum the values of all six components and multiply the total by 20/6. The resulting score ranges from 20 to 80. A score of 44 or above indicates anxiety, while a score below 44 is considered no or low anxiety^21^.

### Data collection tools and procedures

Data were collected using a semi-structured, interviewer-administered questionnaire and standardized assessment tools through face-to-face interviews and medical chart reviews. Preoperative anxiety was assessed before surgery, after admission and prior to anesthetic induction, to capture baseline anxiety levels unaffected by anesthesia or postoperative pain, using the short-form State Anxiety Inventory, which was translated through a forward–backward translation approach and culturally adapted through pretesting to ensure clarity and contextual relevance. Postoperative pain severity was assessed 24 h after surgery (postoperative day one) using the Numerical Rating Scale (NRS), where 0 indicates no pain and 10 indicates the worst imaginable pain. This time point was chosen to capture clinically meaningful pain intensity after adequate exposure to analgesic interventions while minimizing residual anesthetic effects.

At the same time point, patient satisfaction with postoperative pain management was assessed, enabling patients to evaluate the effectiveness and adequacy of pain management during the peak postoperative period while reducing recall bias.

The satisfaction tool was adapted from the Assessment of Patient Satisfaction with Acute Pain Management Service (APMS)^22^, the internal consistency of the patient satisfaction scale was assessed using Cronbach’s alpha, yielding a value of 0.82, indicating good reliability in this study population. The questionnaire comprised three sections: socio-demographic characteristics, clinical related characteristics, and patient satisfaction. Data were collected by two trained third-year anesthesia students using standardized procedures across all participating centers to ensure data consistency and quality.

### Data quality control and management

To ensure the reliability of the data collected in the study, the questionnaire was initially prepared in English, then translated into the local language (Amharic), and subsequently back-translated into English to verify consistency. A two-day training session was conducted for data collectors on data collection techniques, followed by a pretest involving 5% of the sample size at DTCSH before the actual data collection began; the samples used in the pretest were not included in the study. Data were verified for accuracy, clarity, and completeness, and any identified errors or omissions were promptly corrected on a daily basis.

### Data processing and analysis

The collected data was cleaned, coded, and checked for completeness before being entered into STATA version 17 for analysis. Descriptive statistics were employed to characterize the data and assess the distribution of study variables. The association between independent factors and the outcome variable was evaluated using the chi-squared test, as well as bi-variable and multi-variable logistic regression. Crude and adjusted odds ratios, along with 95% confidence intervals, were calculated to estimate the strength of these associations. Variables with a *p*-value less than 0.2 from the bi-variable logistic regression were included in the multi-variable logistic regression. Ultimately, variables with a *p*-value less than 0.05 from the adjusted odds ratio were considered significant factors. The data was presented through narration, tables, and figures.

Interaction effects were not formally explored in the final analysis. The primary objective of the study was to identify independent factors associated with patient satisfaction, and the available sample size limited the ability to perform reliable interaction analyses across multiple subgroups. Future studies with larger sample sizes could explore potential interaction effects between perioperative variables in greater detail.

Pain severity was not included in the primary multivariable logistic regression model because it was conceptualized as an intermediate (mediating) variable on the causal pathway between perioperative care factors and patient satisfaction. Additionally, patient satisfaction was assessed using the Acute Pain Management Service (APMS) patient satisfaction tool, which incorporates pain-related dimensions such as pain intensity and relief. Including pain severity as an independent variable would therefore introduce conceptual and statistical overlap with the outcome (mathematical coupling) and may lead to overadjustment bias. To evaluate the robustness of the findings, a sensitivity analysis was performed by including pain severity in the final model.

### Ethical considerations

The study protocol was reviewed and approved by the Community-Based Education Office, College of Health Sciences, Debre Tabor University, under ethical approval number CHS/358/2025. The study was conducted in accordance with the principles of the Declaration of Helsinki. Written and verbal informed consent was obtained from all participants prior to data collection. Participants were informed of their right to withdraw at any time without any consequences. Confidentiality was maintained by using anonymized codes and restricting data access to the research team.

## Results

### Socio-demographic and preoperative clinical characteristics of study participants

The study population was predominantly young adults aged 18–39 years (272, 64.3%). Males constituted the majority of participants (293, 69.3%), and most respondents were from rural areas (264, 62.4%). Regarding education, the largest groups were those with secondary education (98, 23.2%) and those unable to read and write (92, 21.8%). Farmers represented the most common occupational group (168, 39.7%), and majority participants were married (254, 60.1%). Clinically, the majority were classified as ASA I (274, 64.8%), and more than half underwent elective surgery (238, 56.3%). A substantial proportion had no history of alcohol intake (266, 62.9%) and no history of smoking (402, 95.0%). Nearly half of the patients reported high preoperative anxiety (195, 46.1%), while most had no history of chronic pain (388, 91.7%). Preoperative analgesia was administered to 177 patients (41.8%) (Table 1).

**Table 1:** Socio-demographic and preoperative clinical characteristics of study participants at Comprehensive Specialized Hospitals in the ‘*REDACTED*’ of Northwest Ethiopia, 2025 (*N* = 423).

Variables	Category	Frequency (%)	Satisfaction	
			Satisfied (*n*% )	Not satisfied (*n*% )
Age (years)	18–39	272 (64.30)	218 (80.15)	54 (19.85)
	40–64	128 (30.26)	105 (82.03)	23 (17.97)
	≥ 65	23 (5.44)	13 (56.52)	10 (43.48)
Sex	Male	293 (69.27)	232 (79.18)	61 (20.82)
	Female	130 (30.73)	104 (80.00)	26(20.00)
Residence	Rural	264 (62.41)	209 (79.17)	55 (20.83)
	Urban	159 (37.59)	127 (79.87)	32 (20.13)
Educational status	Unable to read and write	92 (21.75)	75 (81.52)	17 (18.48)
	Able to read and write	83 (19.62)	65 (78.31)	18 (21.69)
	Primary school	84 (19.86)	63 (75.00)	21 (25.00)
	Secondary	98 (23.17)	82 (83.67)	16 (16.33)
	College& above	66 (15.60)	51 (77.27)	15 (22.73)
Occupation status	Student	76 (17.97)	57 (75.00)	19 (25.00)
	Unemployed	12 (2.84)	10 (83.33)	2 (16.67)
	Farmer	168 (39.72)	133 (79.17)	35 (20.83)
	House wife	49 (11.58)	41 (83.67)	9 (16.33)
	Employed	67 (15.84)	52 (77.61)	15 (22.39)
	Merchant	49 (11.58)	42 (85.71)	7 (14.29)
	Retired	2 (0.47)	1 (50.00)	1 (50.00)
Marital status	Single	135 (31.91)	97 (71.85)	38 (28.15)
	Married	254 (60.05)	217 (85.43)	37 (14.57)
	Separated	15 (3.55)	11 (73.33)	4 (26.67)
	Divorced	8 (1.89)	5 (62.50)	3 (37.50)
	Widowed	11 (2.60)	6 (54.55)	5 (45.45)
ASA status	ASA I	274 (64.78)	219 (79.93)	55 (20.07)
	ASA II	132 (31.21)	106 (80.30)	26 (19.70)
	ASA III	17 (4.02)	11 (64.71)	6 (35.29)
Urgency of surgery	Elective	238 (56.26)	200 (84.03)	38 (15.97)
	Emergency	185 (43.74 )	136 (73.51)	49 (26.49)
History of alcohol intake	Yes	157 (37.12)	122 (77.71)	35 (22.29)
	No	266 (62.88)	214 (80.45)	52 (19.55)
History of smoking	Yes	21 (4.96)	16 (76.19)	5 (23.81)
	No	402 (95.04)	320 (79.60)	82 (20.40)
Preoperative anxiety	No/low anxiety (≤ 44)	228 (53.90)	200 (87.72)	28 (12.28)
	High anxiety (> 44)	195 (46.10)	136 (69.74)	59 (30.26)
History of chronic pain	Yes	35 (8.27)	29 (74.29)	6 (25.71)
	No	388 (91.73)	310 (79.90)	78 (20.10)
Preoperative analgesia given	Yes	177 (41.84)	147 (83.05)	30 (16.95)
	No	246 (58.16)	189 (76.83)	57 (23.17)

ASA, American Society of Anesthesiologist; *n*, Frequency, %, Percentage.

### Intraoperative and postoperative characteristics of study participants

The study primarily included internal fixation procedures (198, 46.8%), followed by infection-related procedures (73, 17.3%) and implant removal (60, 14.2%). Neuraxial anesthesia was the most frequently administered anesthetic technique (261, 61.7%), with peripheral nerve blocks also commonly used (143, 33.8%). In contrast, general anesthesia was applied in a small proportion of cases (17, 4.0%). Most patients received intraoperative analgesia through the anesthesia itself (271, 64.1%), and the majority of surgeries had an intermediate duration (1–3 h) (298, 70.5%). Postoperatively, non-opioid–based analgesia was the most commonly utilized method (167, 39.5%), followed by peripheral nerve block-based postoperative analgesia (118, 27.9%). In comparison, opioid-based postoperative analgesia was used less frequently (43, 10.2%) (Table 2).

**Table 2:** Intra-operative and postoperative characteristics of study participants at Comprehensive Specialized Hospitals in the ‘*REDACTED*’ of Northwest Ethiopia, 2025 (*N* = 423).

Variables	Category	Frequency (%)	Satisfaction	
			Satisfied (*n* %)	Not satisfied (*n* %)
Type of procedure	Internal fixation	198 (46.81)	155 (78.28)	43 (21.72)
	External fixation	45 (10.64)	36 (80.00)	9 (20.00)
	Infection-related procedures	73 (17.26)	54 (73.97)	19 (26.03)
	Implant removal	60 (14.18)	54 (90.00)	6 (10.00)
	Arthroplasty/amputation	47 (11.11)	37 (78.72)	10 (21.28)
Types of anesthesia given	Neuraxial anesthesia	261 (61.70)	208 (78.93)	55 (21.07)
	Peripheral nerve block (PNB)	143 (33.81)	118 (82.52)	25 (15.48)
	General anesthesia	17 (4.02)	10 (58.82)	7 (41.18)
	Neuraxial and PNB combined	2 (0.47)	2 (100.00)	0
Intraoperative analgesia	Anesthesia itself	271 (64.07)	227 (83.76)	44 (16.24)
	Multimodal (opioid + non-opioid)	13 (3.07)	9 (69.23)	4 (30.77)
	Non-opioid systemic analgesia	104 (24.57)	80 (76.92)	24 (23.18)
	Opioid systemic analgesia	35 (8.27)	20 (57.14)	15 (42.86)
Duration of the procedure	Short surgery (≤ 1 h)	77 (18.20)	69 (89.61)	8 (10.39)
	Intermediate surgery (1–3 h)	298 (70.45)	230 (77.18)	68 (22.82)
	Long surgery (> 3 h)	48 (11.35)	37 (77.08)	11 (22.92)
Postoperative analgesia	Non-opioid and Opioid analgesia	95 (22.46)	75 (78.95)	20 (21.05)
	Non-opioid based analgesia	167 (39.48)	129 (77.25)	38 (22.75)
	Opioid-based analgesia	43 (10.17)	26 (60.47)	17 (39.53)
	Peripheral nerve block-based pop analgesia	118 (27.90)	106 (89.83)	12 (10.17)

GA, General anesthesia; PNB, Peripheral nerves block; POP, Postoperative pain, *n*, Frequency, %, Percentage.

### Patient satisfaction in postoperative pain management and postoperative pain severity

**Fig. 1:**
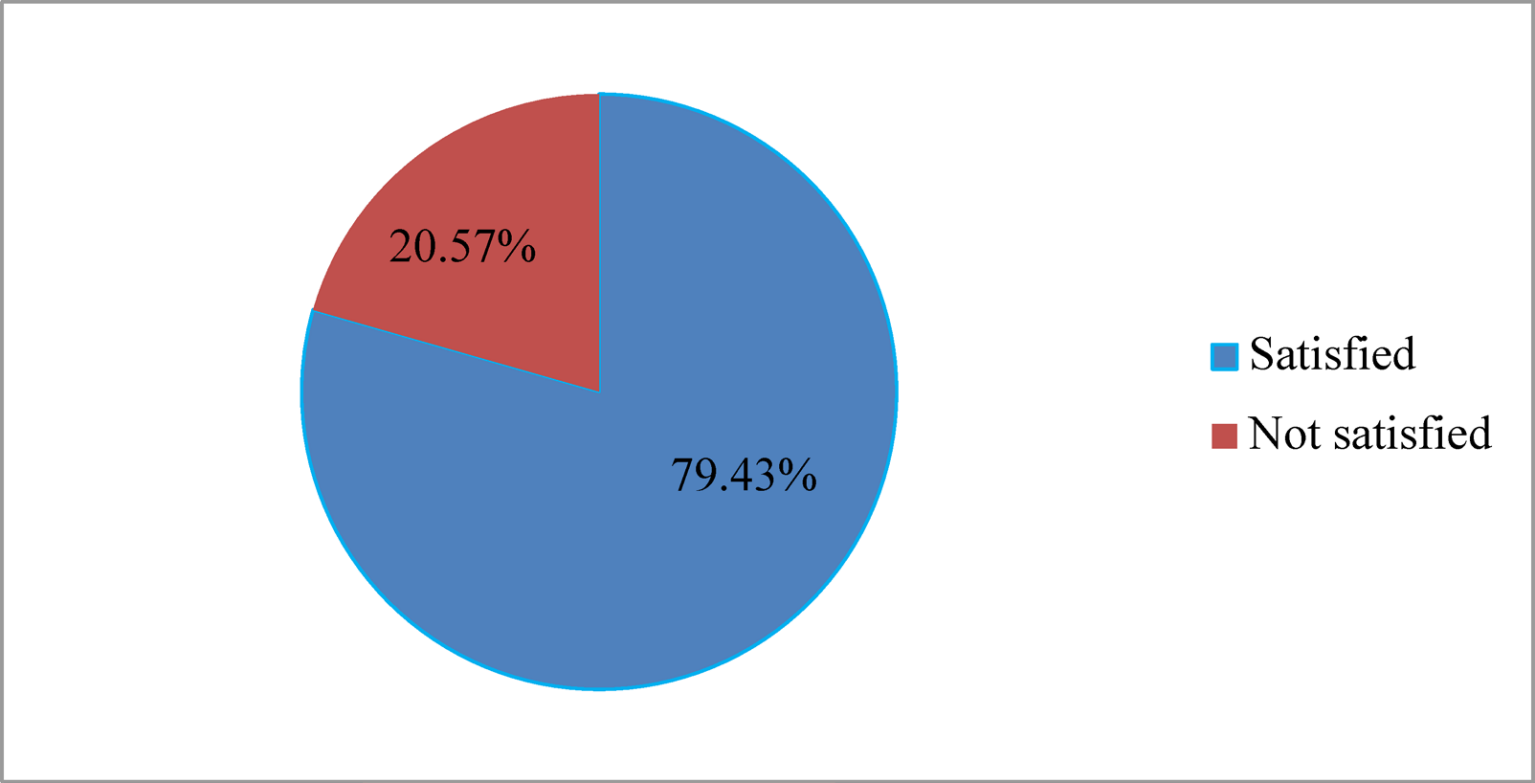
Patient satisfaction with postoperative pain management among adult patients who underwent orthopedic surgery at Comprehensive Specialized Hospitals in the Amhara region of Northwest Ethiopia in 2025 (*N* = 423).

**Fig. 2:**
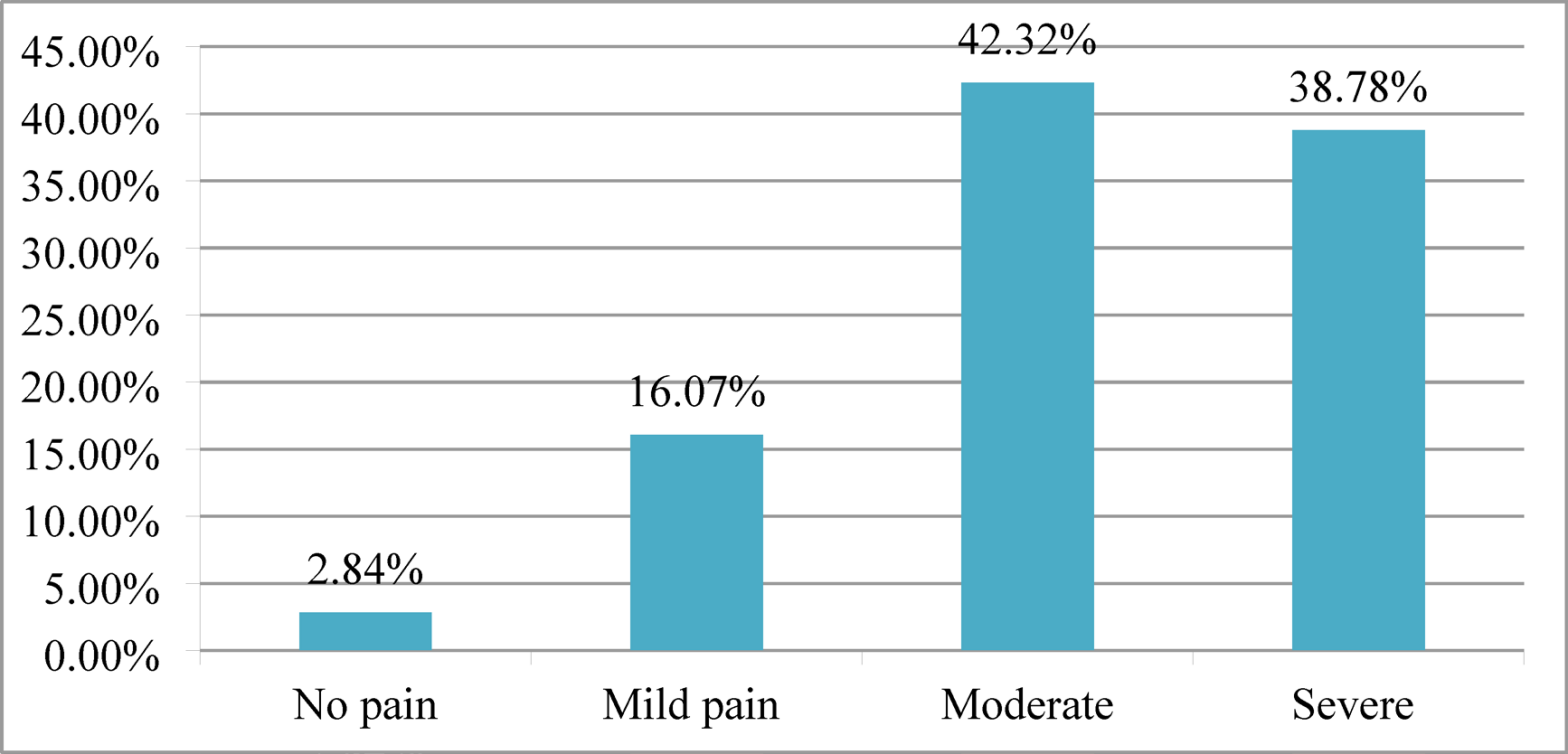
Patient postoperative pain severity among adult patients who underwent orthopedic surgery at Comprehensive Specialized Hospitals in the Amhara region of Northwest Ethiopia in 2025 (*N* = 423).

Patient satisfaction with postoperative pain management in this study was 336 (79.43%), with a 95% confidence interval (CI) of (75.30, 83.03) (Fig. 1). Out of the total participants, 12 (2.84%) reported no pain, 66 (16.07%) experienced mild pain, 179 (42.32%) had moderate pain, and 164 (38.77%) experienced severe pain postoperatively (Fig. 2). Overall, the majority of patients 343 (81.09%) reported moderate-to-severe pain after surgery.

### Factors associated with satisfaction in postoperative pain management

In the bi-variable logistic regression analysis, eleven independent variables were selected for multi-variable logistic regression analysis, using a *p*-value threshold of < 0.2 as a reference. The variables included age, type of procedure, marital status, ASA status, urgency of surgery, preoperative anxiety, preoperative analgesia, intraoperative analgesia, duration of the procedure, and postoperative analgesia. The multi-variable logistic regression revealed that the following variables were significantly associated with satisfaction in postoperative pain management: urgency of surgery, preoperative anxiety, preoperative analgesia, duration of the procedure, and peripheral nerve block-based postoperative analgesia (Table 3).

**Table 3:** Multivariable logistic regression analysis identified factors associated with patient satisfaction in the management of postoperative pain among adults who underwent orthopedic surgery at Comprehensive Specialized Hospitals in the ‘*REDACTED*’ of Northwest Ethiopia, 2025 (*N* = 423).

Variables	Satisfaction		COR (95% CI)	AOR (95% CI)	*p*-value	
	Satisfied (*n* %)	Not satisfied (*n* %)				
Age						
18–39	218 (64.88)	54 (62.07)	3.11 (1.29, 7.46)	1.80 (0.62, 5.25)		0.28
40–64	105 (31.25)	23 (26.44)	3.51 (1.37, 8.99)	1.81 (0.60, 5.47)		0.29
≥ 65	13 (3.87)	10 (11.49)	1	1		
Type of procedure						
Internal fixation	155 (46.13)	43 (49.43)	1	1		
External fixation	36 (10.71)	9 (10.34)	1.11 (0.49, 2.48)	1.73 (0.65, 4.62)		0.27
Infection-related procedures	54 (16.07)	19 (21.84)	0.79 (0.42, 1.47)	1.35 (0.61, 3.07)		0.45
Implant/hardware removal	54 (16.07)	6 (6.90)	2.50 (1.01, 6.19)	1.55 (0.52, 4.60)		0.42
Arthroplasty/amputation	37 (11.01)	10 (11.49)	1.03 (0.47, 2.23)	1.21 (0.45, 3.24)		0.70
Marital status						
Single	97 (28.87)	38 (43.68)	1.53 (0.35, 6.73)	1.37 (0.27, 7.07)		0.70
Married	217 (64.58)	37 (42.53)	3.52 (0.81, 15.35)	4.10 (0.80, 20.97)		0.90
Separated	11 (3.27)	4 (4.60)	1.65 (0.26, 10.31)	2.99 (0.36, 25.02)		0.31
Divorced	5 (1.49)	3 (3.45)	1	1		
Widowed	6 (1.79)	5 (5.75)	0.72 (0.11, 4.62)	0.91 (0.12, 7.12)		0.93
ASA status						
ASA I	219 (65.18)	55 (63.22)	2.17 (0.77, 6.13)	2.92 (0.85, 10.02)		0.09
ASA II	106 (31.55)	26 (29.89)	2.22 (0.75, 6.57)	2.72 (0.78, 2.49)		0.12
ASA III	11 (3.27)	6 (6.90)	1	1		
Urgency of surgery						
Elective	200 (59.52)	38 (43.68)	1.90 (1.18, 3.05)	2.92 (1.58, 5.40)		0.001
Emergency	136 (40.48)	49 (56.32)	1	1		
Preoperative anxiety						
No/low anxiety (≤ 44)	200 (59.52)	28 (32.18)	3.10 (1.88, 5.11)	2.98 (1.67, 5.34)		0.000
High anxiety (> 44)	136 (40.48)	59 (67.82)	1	1		
Preoperative analgesia given						
Yes	147 (43.75)	30 (34.48)	1.48 (0.90, 2.42)	2.39 (1.29, 4.40)		0.005
No	189 (56.25)	57 (65.52)	1	1		
Intraoperative analgesia						
Anesthesia itself	227 (67.56)	44 (50.57)	1	1		
Opioid and Non-opioid	9 (2.68)	4 (4.60)	1.61 (0.19, 13.35)	1.61 (0.16, 16.01)		0.68
Non-opioid systemic analgesia	80 (23.81)	24 (27.59)	0.53 (0.28, 0.99)	1.00 (0.44, 2.29)		0.99
Opioid systemic analgesia	20 (5.95)	15 (17.24)	0.24 (0.11, 0.54)	0.41 (0.16, 1.08)		0.07
Duration of the procedure						
Short surgery (≤ 1 h)	69 (20.54)	8 (9.20)	2.54 (0.95, 6.93)	3.71 (1.11, 12.37)		0.03
Intermediate surgery (1–3 h)	230 (68.45)	68 (78.16)	1.01 (0.49, 2.08)	1.24 (0.50, 3.07)		0.65
Long surgery (> 3 h)	37 (11.01)	11 (12.64)	1	1		
Postoperative analgesia						
Non-opioid and opioid analgesia	75 (22.32)	20 (22.99)	1.10 (0.60, 2.04)	1.29 (0.62, 16.01)		0.50
Non-opioid based analgesia	129 (38.39)	38 (43.68)	1	1		
Opioid-based analgesia	26 (7.74)	17 (19.54)	0.45 (0.22, 0.92)	0.62 (0.25, 1.50)		0.29
Peripheral nerve block-based pop analgesia	106 (31.55)	12 (13.79)	2.60 (1.29, 5.23)	2.98 (1.27, 7.02)		0.012

1, Reference; ASA, American Society of Anesthesiologist; COR, Crude Odds Ratio; AOR, Adjusted Odds Ratio; CI, Confidence Interval; POP, Postoperative Pain; *n*, Frequency.

In a sensitivity analysis including pain severity, effect estimates were attenuated but remained directionally consistent, while pain severity was significantly associated with satisfaction.

Patients who underwent elective surgery were almost three times more likely to be satisfied with postoperative pain management compared with those undergoing emergency surgery (AOR = 2.92, 95% CI 1.58–5.40). Patients with no or low preoperative anxiety had about threefold higher odds of satisfaction than those with high anxiety (AOR = 2.98, 95% CI 1.67–5.34) (Table 2).

Receiving preoperative analgesia was significantly associated with higher patient satisfaction with postoperative pain management. Patients who received preoperative analgesia had more than twice the odds of reporting satisfaction compared with those who did not receive it (AOR = 2.39, 95% CI 1.29–4.40). Similarly, shorter surgical duration (≤ 1 h) was associated with higher satisfaction levels. Patients who underwent short surgical procedures had nearly four times higher odds of reporting satisfaction compared with those who underwent longer procedures (> 3 h) (AOR = 3.71, 95% CI 1.11–12.37). In addition, the use of peripheral nerve block-based postoperative analgesia was significantly associated with higher patient satisfaction. Patients who received peripheral nerve block-based analgesia had almost three times higher odds of being satisfied with pain management compared with those who received non-opioid–based analgesia (AOR = 2.98, 95% CI 1.27–7.02) (Table 3).

## Discussion

This multicenter cross-sectional study assessed postoperative pain management satisfaction and its determinants among adult orthopedic patients in comprehensive specialized hospitals of Northwest Ethiopia. The overall satisfaction level with postoperative pain management was 79.43% (95% CI 75.30–83.03). Several factors were independently associated with satisfaction, including urgency of surgery, preoperative anxiety, preoperative analgesia, duration of surgery, and use of peripheral nerve block-based postoperative analgesia.

### Magnitude of patient satisfaction with postoperative pain management

In the present study, nearly four-fifths of orthopedic patients reported satisfaction with postoperative pain management. This finding is comparable with studies conducted in Nigeria (82.8%)^23^ and Spain (81.90%)^4^. This similarity may be attributed to the use of comparable Likert-scale–based satisfaction measurement tools, similar patient profiles dominated by ASA I–II and elective orthopedic procedures, and the widespread use of regional and multimodal analgesia techniques. In particular, the frequent application of neuraxial anesthesia and peripheral nerve blocks—shown in the present study to significantly improve satisfaction—has also been reported in the Nigerian and Spanish studies.

Additionally, patient satisfaction does not necessarily correspond directly with pain intensity alone. Satisfaction is a multidimensional construct influenced by factors such as patient expectations, communication with healthcare providers, responsiveness of staff, and perceived efforts to relieve pain. In many clinical settings, patients may report satisfaction even when experiencing moderate or severe pain if they perceive that healthcare providers are attentive and actively attempting to manage their discomfort. In the present study, this apparent paradox was supported by our data, where among the 343 patients (81.1%) who experienced moderate-to-severe postoperative pain, 74.6% still reported satisfaction with pain management. Further item-level analysis showed that 88% of satisfied patients agreed that healthcare providers did everything possible to manage their pain, indicating that perceived provider effort and attentiveness were key determinants of satisfaction rather than complete pain relief. This finding highlights that pain intensity and satisfaction are related but not mutually exclusive outcomes. This phenomenon may partly explain the apparent discrepancy between relatively high levels of reported postoperative pain and the high satisfaction observed in this study.

However, the satisfaction level observed in this study is higher than reports from Ethiopia (Gondar (72.2%)^24^, and Addis Ababa (74.5%)^1^), Addis Ababa (65.0%) and a study from Nigeria (68.4%), Tanzania (74.3%)^25^, India (69%)^9^, and Jordan (66.6%)^26^. The higher postoperative pain management satisfaction observed in the present study compared with reports from Gondar and Addis Ababa, Nigeria, Tanzania, India, and Jordan may be explained by differences in perioperative care and study populations. Unlike the Gondar and Addis Ababa studies, which reported lower satisfaction and limited use of regional analgesia, the current study demonstrated widespread utilization of neuraxial anesthesia and peripheral nerve block-based postoperative analgesia, both of which were independently associated with higher satisfaction. Similarly, studies from Nigeria, Tanzania, and India included mixed surgical populations with a higher proportion of emergency procedures and greater reliance on systemic analgesics, factors known to negatively influence pain control and patient satisfaction. The Jordanian study, which reported the lowest satisfaction, also documented high pain prevalence and minimal preoperative analgesia and anxiety management. In contrast, the present study included a higher proportion of elective surgeries, greater use of preoperative analgesia, lower preoperative anxiety, and shorter surgical duration, all of which significantly contributed to improved satisfaction. Methodological differences in satisfaction assessment tools and cut-off thresholds across studies may have further influenced the observed variations.

Another possible explanation for the relatively high satisfaction level observed in this study, despite reports of moderate to severe postoperative pain among some patients, may relate to cultural and contextual factors. In many low- and middle-income settings, including Ethiopia, patients often have modest expectations regarding postoperative pain relief and may perceive pain as an inevitable or unavoidable part of surgery. Furthermore, cultural norms of respect and gratitude toward healthcare providers may lead patients to report higher satisfaction with the care they receive, even when pain is not fully controlled. This cultural perspective, together with the influence of patient expectations and provider–patient communication, provides a more nuanced, data-informed explanation for the coexistence of substantial postoperative pain and high satisfaction observed in this study.

Conversely, the finding is lower than studies conducted in Ethiopia (Mekele (90.30%)^8^, Addis Ababa (92.2%)^27^), Ghana (96%)^28^, and Beirut (85%)^29^. These higher satisfaction rates may be explained by differences in healthcare settings and pain management practices. Studies from Addis Ababa, Ghana, and Beirut were conducted in better-resourced tertiary hospitals where standardized pain management protocols, routine preemptive analgesia, multimodal approaches, and wider use of regional techniques are more common. In contrast, the current study was conducted in resource-limited settings, with a lower proportion of patients receiving preoperative analgesia (41.8%) and peripheral nerve block-based postoperative analgesia (27.9%). Additionally, the present study included a substantial proportion of emergency surgeries (43.7%) and patients with high preoperative anxiety (46.1%), both of which were independently associated with lower satisfaction, whereas studies reporting higher satisfaction predominantly involved elective procedures with better preoperative counseling. Furthermore, Variations in satisfaction measurement tools, scale formats, and cutoff values across studies may also contribute to differences in reported satisfaction rates. Because different instruments capture distinct dimensions of patient experience and apply different classification thresholds, direct comparisons between studies conducted in different countries should be interpreted cautiously.

It is also important to note that comparisons of satisfaction rates across studies should be interpreted carefully because measurement approaches differ substantially. Various studies use different patient satisfaction instruments, Likert-scale formats, or dichotomization thresholds to define “satisfied” patients. These methodological differences can influence reported satisfaction levels and limit the direct comparability of findings between countries or healthcare systems.

### Factors associated with satisfaction in postoperative pain management

Undergoing elective surgery was significantly associated with higher patient satisfaction with postoperative pain management, with patients who underwent elective surgery having nearly three times higher odds of satisfaction compared with those who underwent emergency surgery. This finding is consistent with studies from Ethiopia^1^, India^9^, and Nigeria^30^. This may be due to elective care, which allows for preoperative counseling, expectation setting, and planned multimodal analgesia. These factors positively influence patients’ perceptions of pain control and care quality^31^. Elective patients often report higher satisfaction, even when pain persists, largely because of the adequate information and structured analgesic protocols they receive^9,31^.

Patients with no or low preoperative anxiety had approximately threefold higher odds of satisfaction compared with those with high anxiety. This finding aligns with studies conducted in Turkey^32^. High anxiety may amplify pain perception through neuroendocrine stress responses, leading to increased analgesic requirements and dissatisfaction^33,34^. This highlights the importance of preoperative psychological assessment and counseling as part of comprehensive pain management.

Receiving preoperative analgesia was significantly associated with higher patient satisfaction with postoperative pain management, with patients who received preoperative analgesia having more than twice the odds of reporting satisfaction compared with those who did not receive it. This finding is consistent with studies from Brazil^33^, United Kingdom (Meta-analysis)^35^. Preoperative analgesia may reduce central sensitization and postoperative pain intensity, thereby enhancing patient comfort and satisfaction^36,37^. This supports the growing evidence favoring preemptive and multimodal analgesic approaches.

Short surgical procedures (≤ 1 h) were significantly associated with higher satisfaction compared with prolonged surgeries (> 3 h). This finding has been supported in a study from USA^38^. Longer surgical duration is often associated with increased tissue trauma, higher postoperative pain intensity, and delayed recovery, which may negatively affect patient satisfaction^39,40^.

Patients who received peripheral nerve block-based postoperative analgesia were almost three times more likely to be satisfied compared with those receiving non-opioid–based analgesia alone. This finding is consistent with studies from Gondar (Ethiopia)^24^, India^41^. Peripheral nerve blocks provide site-specific analgesia, reduce systemic analgesic requirements, and minimize opioid-related side effects, which may contribute to higher satisfaction levels^37,42,43^.

### Implications for clinical practice

The findings of this study underscore the importance of preoperative anxiety management, planned analgesic strategies, and wider utilization of peripheral nerve blocks to improve patient satisfaction with postoperative pain management. Expanding regional anesthesia services may contribute to improving patient satisfaction and pain management outcomes in resource-limited settings.

### Limitation of the study

This study has several limitations that should be considered when interpreting the findings. First, the use of consecutive sampling may limit the generalizability of the results to all orthopedic surgical patients. Second, the cross-sectional study design restricts the ability to establish causal relationships between identified factors and patient satisfaction with postoperative pain (POP) management and does not capture potential changes in pain experiences over time. In addition, satisfaction was assessed at a single time point within 24 h after surgery, and satisfaction levels may differ if assessed later during hospitalization or at discharge as patients progress through recovery and experience additional postoperative care. Because satisfaction was measured using interviewer-administered questionnaires, there is a possibility of social desirability bias, where participants may have provided more favorable responses regarding the care they received. Furthermore, interviewer bias cannot be completely ruled out despite the training provided to data collectors. Although assessing satisfaction within 24 h helps minimize memory-related errors, some degree of recall bias may still be present. The analysis also did not adjust for potential clustering effects across the participating hospitals, which may influence estimates if institutional practices differ. Finally, the exclusion of patients with known psychiatric disorders and those unable to communicate may limit the generalizability of the findings to these patient populations.

## Conclusion and recommendations

Postoperative pain management satisfaction among adult orthopedic patients was moderately high, with satisfaction significantly influenced by elective surgery, lower preoperative anxiety, preoperative analgesia, shorter surgical duration, and the use of peripheral nerve block-based postoperative analgesia. These findings highlight that both patient-related factors and perioperative care practices are important factors associated with patient satisfaction. To enhance patient-centered outcomes, healthcare facilities should prioritize routine preoperative anxiety assessment and counseling, the systematic administration of preoperative analgesia, and the consistent use of peripheral nerve block-based analgesia. In resource-limited settings, maintaining landmark-based peripheral nerve block techniques where advanced equipment is unavailable, along with ongoing provider training may support safe and effective implementation. In addition, careful surgical planning to prioritize elective procedures and minimize operative time may be beneficial for postoperative pain control and overall patient satisfaction, particularly in resource-limited hospital settings.

## Data Availability

The datasets generated and/or analyzed are not publicly available due to confidentiality but are available from the corresponding author upon reasonable request.

## Abbreviations

AOR: Adjusted odds ratio
ASA: American Societies of Anesthesiologist
CI: Confidence interval
COR: Crude odds ratio
DTCSH: Debre Tabor Comprehensive Specialized Hospital
KM: Kilo meter
POP: Postoperative pain
PNB: Peripheral nerve block
TASH: Tikur Anbessa Specialized Hospital
TGSH: Tibebe Gion Specialized Hospital
UoGCSH: University of Gondar Comprehensive Specialized Hospital
